# A Comparison of Three Holistic Approaches to Health: One Health, EcoHealth, and Planetary Health

**DOI:** 10.3389/fvets.2017.00163

**Published:** 2017-09-29

**Authors:** Henrik Lerner, Charlotte Berg

**Affiliations:** ^1^Department of Health Care Sciences, Ersta Sköndal Bräcke University College, Stockholm, Sweden; ^2^Department of Animal Environment and Health, SLU Swedish University of Agricultural Sciences, Skara, Sweden

**Keywords:** concept of health, ecology, ecosystems, interdisciplinarity, philosophy of medicine, medicine, value, veterinary medicine

## Abstract

Several holistic and interdisciplinary approaches exist to safeguard health. Three of the most influential concepts at the moment, One Health, EcoHealth, and Planetary Health, are analyzed in this paper, revealing similarities and differences at the theoretical conceptual level. These approaches may appear synonymous, as they all promote the underlying assumption of humans and other animals sharing the same planet and the same environmental challenges, infections and infectious agents as well as other aspects of physical—and possibly mental—health. However, we would like to illuminate the differences between these three concepts or approaches, and how the choice of terms may, deliberately or involuntary, signal the focus, and underlying values of the approaches. In this paper, we have chosen some proposed and well-known suggestions of definitions. In our theoretical analysis, we will focus on at least two areas. These are (1) the value of the potential scientific areas which could be included and (2) core values present within the approach. In the first area, our main concern is whether the approaches are interdisciplinary and whether the core scientific areas are assigned equal importance. For the second area, which is rather wide, we analyze core values such as biodiversity, health, and how one values humans, animals, and ecosystems. One Health has been described as either a narrow approach combining public health and veterinary medicine or as a wide approach as in the wide-spread “umbrella” depiction including both scientific fields, core concepts, and interdisciplinary research areas. In both cases, however, safeguarding the health of vertebrates is usually in focus although ecosystems are also included in the model. The EcoHealth approach seems to have more of a biodiversity focus, with an emphasis on all living creatures, implying that parasites, unicellular organisms, and possibly also viruses have a value and should be protected. Planetary Health, on the other hand, has been put forward as a fruitful approach to deal with growing threats in the health area, not least globally. We conclude that there are actually important differences between these three approaches, which should be kept in mind when using any of these terms.

## Introduction

Several holistic and interdisciplinary approaches that work with the human–animal–environment interface exist in order to safeguard health. Three of the most influential concepts at this moment, One Health, EcoHealth, and Planetary Health, are analyzed in this paper, revealing similarities and differences at the theoretical, conceptual level. Of these three concepts, Planetary Health is a more recent and therefore possibly less developed concept, and it may hence appear somewhat arbitrary to choose this concept and compare it to the two more well-established ones. However, based on the fact that Planetary Health has rapidly become an approach mentioned in very well-renowned and high-ranked global publications and furthermore seems to attract attention among politically influential groups we still find it highly relevant to include this concept in the discussion on an equal basis.

Someone approaching these concepts from the outside may easily perceive these concepts as relatively synonymous, as they all promote the underlying assumption of humans and other animals sharing the same planet, and to large extent the same habitats and with this the same environmental challenges, infections, and infectious agents as well as other aspects of physical—and possibly mental—health. Since a couple of years back, there has been an ongoing effort to merge at least two of these approaches, namely One Health and EcoHealth (1, 2). Among the papers promoting a merge some differences are acknowledged although downplayed, issues that might question the idea that the two approaches are synonymous. Also during this period, at least one new approach, Planetary health, has been proposed as an alternative to the other two (3).^1^ We believe that a more thorough analysis of the differences between the approaches is needed for two different reasons. Either the analysis gives an argument to halter the merge or the analysis might help keeping the diversity in a merged approach.

It should be kept in mind that there are no universal, agreed definitions of any of these three approaches (6, 7). Attempts have been made to pinpoint the central aspects of each of these, but these aspects are not centrally agreed on and different people, within different professions, and with different backgrounds, may use the approaches differently. Gibbs (7), for example, argues that definitions of One Health seem to reflect the aim of the organization proposing it. In this paper, we have chosen some proposed and well-known suggestions of definitions. In our theoretical analysis, we will mainly focus on two areas. These are (1) potential scientific areas which could be included and (2) core values present within the approach. In the first area, our main concern is how wide or narrow the view on interdisciplinarity is (i.e., which and how many scientific fields included) and whether the core scientific areas are assigned equal importance. For the second area, which is rather wide, we analyze core values such as biodiversity, health, and how one values humans, animals, and ecosystems.

The aim of this paper is to illuminate the differences between these three approaches, and how the choice of terms may, deliberately or involuntary, signal the focus, and underlying values of the approaches.

## Method

Identifying the demarcation of an interdisciplinary approach is not an easy task. In this study, which is a study in philosophy of science, we have focused on the scientific parts of the approaches and not on the political aspects, although the latter aspect may also warrant deeper analysis by experts in the field. The scientific demarcation of each of these three approaches, which is the question in focus here, relies on underlying values, theories of science, and the scientific fields included. In order to analyze this aspect, one cannot make a straightforward key-word-based bibliometric exercise where any publication in any scientific peer-reviewed journal is read and analyzed. Instead, we have chosen a selective approach where papers and books dealing with theoretical aspects of this field of science, such as theoretical foundation of the approach, how the approach is defined, demarcation of the approach toward other approaches, and possible conflicts of value due to scientific standpoints, are analyzed philosophically. It is based on active searches in several databases and reading of key publications, thorough reading of reference lists, web pages, and newsletters dedicated to the approaches as well as in-depth discussions with experienced colleagues in the fields studied.

We have focused on published scientific texts which have been regarded as either rich in theoretical substance on these matters or that are influential for the approaches. Often these texts have explicitly mentioned how they demarcate the approach or presented a definition of the approach. However, most of the papers and books that are published within these approaches are focused on other important issues such as policy making, implementation of the approaches, solving practical health problems, or basic scientific research. As a consequence, this review consists of relatively few references.

For each of the three approaches, we have analyzed the definitions applied, described the main contributing sciences, and identified the core values, based on these key publications within each approach.

## One Health

### Definitions of the Approach

One Health has been described as both a narrow and wide approach. The narrow approach is mainly biomedical, focusing on animal, and human health while combining human and veterinary medicine (2, 5, 8). Gibbs (7) has compiled central definitions of One Health. The two widest of these are from the One Health Commission and the One Health Global Network. The One Health Commission defines One Health in the following citation:

One Health is the collaborative effort of multiple health science professions, together with their related disciplines, and institutions—working locally, nationally, and globally—to attain optimal health for people, domestic animals, wildlife, plants, and our environment.^2^

The One Health Global Network defines One Health as an approach:

To improve health and well-being through the prevention of risks and the mitigation of effects of crises that originate at the interface between humans, animals, and their various environments.^3^

For both definitions, focus appears to be on what the One Health Global Network calls a “whole of society” approach where all health sciences and their related disciplines works across borders collaboratively to improve health at an optimal level. Lately, there has been increasing emphasis within the One Health scientific community on the need for widening the One Health concept to encompassing not only human and animal health, but also biodiversity, ecology, climate change, agricultural systems, and various social sciences (8).

### Contributing Sciences

In the narrowest description, One Health combines public health and veterinary medicine (9). In one of the widest approaches as in the wide-spread “umbrella” depiction it includes environmental health, ecology, veterinary medicine, public health, human medicine, molecular, and microbiology, as well as health economics (4). The narrow approach is the oldest one, and has been developed from what was earlier termed “One Medicine.” The approach of One Medicine was mainly developed by veterinarians and physicians (10) and hence very centered on conventional medical issues. The term “One Medicine” was later perceived as too clinical, as the approach to health became widened to also include public health issues and ecology (4, 11). However, a more narrow interpretation of One Health, rather similar to that of One Medicine, can still sometimes be found. This narrow approach is, however, not much further discussed in this paper.

As an example, the Manhattan Principles was an ecological approach that expanded the field and belongs to the initial foundation of One Health (1). Also, health is nowadays regarded as something more than just clinical biology, although the outer limits of health as a concept have not been settled within the approach. Critics have pointed out the lack of social sciences including research related to rural development, population dynamics, anthropology, urbanization, and so on within the approach (2). However, there is much effort at the moment to include social scientists, and this aspect was also highlighted at a recent European workshop on One Health (8), together with the already more well-established aspects such as ecology, agricultural systems, food safety and security, and so on.

### Core Values

From our analysis, we find that the core values of the One Health concept still relate to public human health and the health of animals influencing the health of humans. Hence, safeguarding the health, and especially individual health, of vertebrates is usually in focus although ecosystems are also included in the wider model (4). Ecology, microbiology, and biodiversity are nevertheless generally perceived as parts of the core sciences, in their own right. Later publications, such as the report by Keune et al. (8) emphasize the importance of widening the One Health concept also to social sciences and agricultural sciences. It can be argued that two core values of One Health are (a) the respect for scientific specialties whilst emphasizing the need for cooperation between such disciplines and (b) the emphasis application of multidisciplinarity in research and advisory projects.

## EcoHealth

### Definitions of the Approach

EcoHealth has been described as involving the health of humans, animals, and ecosystems, including also environmental sustainability and socioeconomic stability in the framework. In some cases, the EcoHealth approach seems to have more of a biodiversity focus, with an emphasis on all living creatures, implying that parasites, unicellular organisms, and possibly also viruses have a value and should be protected. Waltner-Toews suggests that EcoHealth aims for “sustainable human and animal health and well-being, through healthier ecosystems” [(12), p. 519]. The leading journal of the approach, which has published several papers on these theoretical matters, is the EcoHealth journal. At present, the EcoHealth approach at the EcoHealth Journal website is defined as:

EcoHealth is committed to fostering the health of humans, animals, and ecosystems and to conducting research which recognizes the inextricable linkages between the health of all species and their environments. A basic tenet held is that health and well-being cannot be sustained in a resource depleted, polluted, and socially unstable planet.^4^

### Contributing Sciences

In the start of the journal EcoHealth possible scientific fields that could contribute to the approach was suggested. These were conservation and ecosystem management, veterinary medicine, human medicine, public health practice, rural and urban development, and planning, and more not specified (13). EcoHealth has included more of social science and the humanities than One Health and the approach includes anthropologists with a focus on indigenous people. Indigenous and local knowledge are also acknowledged as a source of knowledge besides the western scientific knowledge (14). Scientific papers could have a more esthetic or essay approach. Among the natural scientists, ecologists seem to have higher influence.

### Core Values

Focus is on the relationship between health, ecosystem, and sustainable development, where the latter is based on equity (1, 6, 13–15). Participation from different sectors in the society such as policymakers, scientists, and those performing the fieldwork are favored (6, 14). That participation is consensus and cooperation-based (15) and aims for action (6). The concept of health is mainly used at the population level of health and is also used as a metaphor (6). Biodiversity is an important value within the idea of sustainability.

## Planetary Health

### Definitions of the Approach

Planetary Health has in recent years been put forward as an alternative to One Health and EcoHealth. The concept has been developed by The Rockefeller Foundation-*Lancet* Commission on planetary health. However, the concept seems to be less interdisciplinary than One Health and EcoHealth and primarily focus on human health, although the environment is acknowledged. In one of the key papers from the commission they state that:

Our definition of planetary health is the achievement of the highest attainable standard of health, well-being, and equity worldwide through judicious attention to the human systems—political, economic, and social—that shape the future of humanity and the Earth’s natural systems that define the safe environmental limits within which humanity can flourish. [(3), p. 1978]

One can clearly see that this definition of planetary health, the Brundtland view of sustainability (16) is present that humans are valued more than other animals or ecosystems. For example, Horton et al. (17) state that Planetary Health is focused on mitigating and responding to threats to human health and well-being, and on the sustainability of the entire human civilization. In addition to this, the authors acknowledge the importance of biodiversity as it is the basis of the natural systems on which humans depend, without discussing the intrinsic values of these ecosystems (17). This approach is extremely anthropocentric, focusing only on human health outcomes. One of the main critiques toward the Brundtland report has been linked to its anthropocentric approach, limiting the discussions to sustainability from human utility perspective, not emphasizing the inherent values of ecological systems and biodiversity (18).

Although also Whitmee et al. (3) to a large extent adopt an anthropocentric approach they bring up effects of ecological changes, climate change, land use alterations, and food production changes in relation to the risks of transmission of zoonotic and vector-borne diseases to humans, thereby involving also animals in the line of thought. Having said this, the health and consequently the welfare of animals for the animals’ own sake is not mentioned, and it is evident that animal health is only perceived as relevant in terms of potential disease transmission to humans, and in terms of their capacity as food production units.

### Contributing Sciences

Approaching the concept of Planetary Health in its most narrow form of interdisciplinarity, Horton et al. (17) clearly state that this field of science involves health professionals, public health practitioners, policy makers, and similar categories. The term “health professionals” in this context only refers to human health professionals, i.e., not veterinarians or other animal health professionals. Neither does this list cover ecologists nor others biologists.

In the key paper from the Rockefeller Foundation and The Lancet (3), the focus is somewhat wider, mentioning a broad spectrum of scientific disciplines such as human medicine, ecology, and other environmental sciences (including climate and biodiversity research), economy, energy, agricultural sciences (including plant and animal production sciences), marine sciences, and more. Hence, these authors appear to acknowledge the need for interdisciplinary collaboration to a larger extent.

### Core Values

From our analysis, the core values within the Planetary Health approach is the health of living and future human generations, applied to individuals, communities, and populations (3). A main goal is equity in health, which is related to socioeconomic, regional, and gender factors (17). Furthermore, the Planetary Health concept requires sustainability, which is in turn based on natural resources and biodiversity (3).

## Discussion

The approach used in this paper was to apply a methodology originating from the field of philosophy science on three interdisciplinary approaches to health among people, animals, and the environment. This approach does have its limitations, as it does not involve a total scrutiny of all publications in these three fields. The choice of papers, books, and informants can always be challenged, but this method is an efficient way of rapidly pinpointing the core values and contributing sciences in a systematic way. Nevertheless, we fully acknowledge that future further and deeper analyses may reveal slightly different results. It should also be mentioned that within the scientific community the values and demarcations of various concepts may change over time, and we would hence like to emphasize that our conclusions only reflect the present state.

Of the three approaches that seemed to be similar, One Health, EcoHealth, and Planetary Health, we have regarded Planetary Health to differ the most regarding how they value humans, animals, and ecosystems (Table 1). This approach is clearly anthropocentric and focuses primarily on human health. In One Health and EcoHealth, humans and animals are more on par. Therefore, we consider Planetary Health as more similar to a concept such as Global Health, than One Health and EcoHealth. One strand of definitions of Global Health is based on a broad collaborative and transnational approach to establish health for all. This “health for all” concerns only humans but is wider than public health (19). The main difference between Global Health and Planetary Health is the emphasis on the need for sustainability based on natural resources in the latter approach.

**Table 1:** Comparison of the three approaches.

		One Health		EcoHealth	Planetary Health	
		Narrow	Wide		Narrow	Wide
Core contributing sciences	Human	Public health	Public health Human medicine Molecular and microbiology Health economics Social sciences	Public health Human medicine Rural and urban development and planning Social sciences Anthropology	Public health Human medicine	Human medicine Economy Energy Natural resources
	Animal	Veterinary medicine	Veterinary medicine	Veterinary medicine	–	Agricultural sciences (including plant and animal production sciences)
	Ecosystem	–	Environmental health Ecology	Conservation and ecosystem management	–	Ecology Other environmental sciences (including climate and biodiversity research) Marine sciences
Knowledge base		Western scientific	Western scientific	Western scientific Indigenous knowledge	Western scientific	Western scientific
Core values	Health	Individual health	Individual and population health	Population health	Individual and population health	Individual and population health
	Groups	Humans Animals	Humans Animals Ecosystems	Humans Animals Ecosystems	Humans	Humans
	Other			Biodiversity Sustainability (for humans, animals, ecosystems)	Sustainability (for humans)	Sustainability (for humans)
Reference		(2, 5, 8, 9)	(4, 7, 8, 11)	(1, 6, 12–15)	(17)	(3)

Regarding the more interdisciplinary and holistic approaches One Health and EcoHealth, both approaches share similarities such as advocating interdisciplinarity, and promoting health. Therefore, some authors argue for a merge of the two approaches despite the existing differences (1, 2). In international reports made by intergovernmental agencies, a practice of treating them as related to each other has already been established [see (20)]. However, there still seem to be some aspects that might differ between the approaches, and we will below discuss the concept of health and the differences in interdisciplinarity. For comparison, we will analyze all three approaches. Finally, as a consequence of the fact that at least One Health and EcoHealth seems to expand in their interdisciplinarity we will also discuss where the outer limits of these approaches might lie.

### Health

One of the most obvious differences is in the view of health. Planetary Health focuses mainly on human health (3), while the other two approaches have a broader perspective. Zinsstag et al. (1) state that One Health mainly treat animal and human health while EcoHealth mainly focuses on the relation between health and ecosystems. The difference between One Health and EcoHealth might be more troublesome to bridge than suggested. Lerner and Berg (4) showed that there are three levels where health can be defined and these are individual level, population level, and ecosystem level. The difference between individual health and the two other levels is similar to the reason why animal ethics and environmental ethics are seen as different from each other. With help from philosophical value theory, one can see that One Health attributes health to individual bearers in the same manner as one strand of animal ethics ascribe values to individual animals and humans, while EcoHealth attributes health to aggregations, systems, and processes similar to when environmental ethics ascribe value to ecosystem processes or species. Could a process have health in the same way as an individual have? Could an ecosystem have health? Or does health become metaphorical in these latter senses, as Charron (6) suggests? On the other hand, if one concerns the human body as an ecosystem, one could rather argue that human individual health should be similar to the ecosystem level. This issue, the relation between and importance of the levels of health, must be solved in order to merge the two approaches.

One initial step may be to decide on treating health as a property of the individual, rather than of the group, population, or ecosystem. This approach is quite possible, without denying the fact that the health of one individual (regardless of species) may of course in many cases directly or indirectly influence the health of others.

Given this, one might want to consider whether there needs to be a similar definition of health for all individuals involved in the merged approach. Should the health promoted be of the same definition for humans, animals, and plants (with relevant adjustments for different kinds of species)? Lerner (21) has shown that this might be possible and several alternatives already exist. For example, the WHO definition of health for humans is currently applied also on animals, especially in organic agriculture. Other categories of health definitions that are applied to both animals and humans are balance theories, health as biological function (such as homeostasis) and health as the ability to realize an individual’s vital goals. When it comes to mental health and its definitions, however, there are still considerable discrepancies in how this scientific field is approached in humans and animals, respectively. Furthermore, the study of health definitions in animals is less thorough than in humans and when one turns to plants or ecosystems even less research and analysis has been carried out. As a conclusion, much philosophical concept analysis still needs to be carried out to find definitions of health suitable to a merged approach [see also (4, 21)].

### Interdisciplinarity

All three approaches are based on multi- or interdisciplinary research. The reason why these approaches have evolved was the understanding that the issues that needed to be solved needed contributions from several disciplines of science [see, for example, Ref. (15)]. To our interpretation, the Planetary Health approach has the narrowest focus on interdisciplinarity, with an emphasis on human health and related research areas. One Health has sometimes been criticized for focusing only on medicine (human and veterinary) (5, 9). Ecohealth seems to be the wider approach accepting more of the disciplines within the humanities and sociology (1) although the One Health approach has during recent years been used in a gradually wider context (4, 8).

Even within a discipline, there is a variety of scientific positions. Within human medicine, for example, some scientists work with microbiology, others with social science aspects and yet others with clinical trials. Therefore, it is reasonable to believe that One Health and EcoHealth could find a demarcation of disciplines that could cooperate in order to solve the problems. This might change due to novel problems, but a core group of disciplines that is wider than today might be easy to agree on (areas neglected or too little mentioned in the debate but could contribute strongly are philosophy and nursing science). Seen from this perspective, it may prove more difficult to incorporate the approach from Planetary Health into a merged approach because of its main focus on humans and human health.

### Outer Boundary

One aspect, which we believe to be an issue, to consider is where the outer boundary should be drawn. A merged approach cannot deal with all aspects of the world without becoming a “theory of everything.” For example, there are voices arguing for a much broader approach called One Welfare, focusing on human, animal, and social welfare including the environment (22–24). The relation of this new wide approach to the merged One Health-EcoHealth approach must be carefully analyzed. In our view, the risks of creating to wide and all-embracing disciplines should not be ignored. To create creative and fruitful interdisciplinary or transdisciplinary research groups and projects, there still has to be basic disciplines to connect between. This can still be the case also for wide concepts such as One Welfare, which must involve several other disciplines in addition to the ones mentioned above. There is a risk of the high number of disciplines involved then resulting in structural problems and conflicts.

At the conceptual level, the relation between the concepts of health and welfare can be seen in different ways depending on how we define them. They may be partially overlapping or more or less independent of each other. In animal welfare science, there has been an emphasis on finding a unifying concept, welfare, which covers all aspects of an animal’s life (25). The problem with this approach is that if some aspects are poor and some are good the overall welfare might be hard to evaluate. Therefore, it is still useful to separate health and welfare conceptually. One can then be able to say that the animal’s health is poor while the welfare is good (26). The same reasoning could be fruitful to apply to the approaches of One Health and One Welfare. One would then be able to focus on different aspects within each field. However, the joint One Welfare approach should not be dismissed until properly evaluated and tested.

## Conclusion

Three of the currently most influential concepts in the area of human, animal, and ecosystem health are One Health, EcoHealth, and Planetary Health. Neither of these concepts have any generally, centrally agreed definitions, and are sometimes handled as almost synonymous, sometimes as overlapping and sometimes as quite distinctly separate. In our analysis, we have found that these concepts have a lot in common but do differ in contributing sciences, core focus, and values, which may influence how they are used and also what signals the choice of term sends. Considering especially the concept of health, the valuing of humans, animals, and ecosystems as well as the view on which disciplines to include within the approach, we conclude that there are actually important differences between these three approaches. This should be kept in mind when using any of these terms or in a process of merging one or more of these approaches together.

## Author Contributions

All authors have planned and contributed in writing the manuscript. All authors have critically reviewed and revised the manuscript and approved the final product.

## Conflict of Interest Statement

The authors declare that the research was conducted in the absence of any commercial or financial relationships that could be construed as a potential conflict of interest.

## Footnotes

^1^See Lerner and Berg (4) for an analysis of another approach, Zoobiquity (5), which has also been introduced during this period but resembles One Health.

^2^https://www.onehealthcommission.org/en/why_one_health/what_is_one_health/ retrieved August 14, 2017.

^3^http://www.onehealthglobal.net/what-is-one-health/ retrieved August 14, 2017.

^4^https://ecohealth.net/en/ retrieved August 14, 2017.
